# de novo MAPT mutation G335A causes severe brain atrophy, 3R and 4R PHF-tau pathology and early onset frontotemporal dementia

**DOI:** 10.1186/s40478-020-00977-8

**Published:** 2020-06-29

**Authors:** Kunie Ando, Lorenzo Ferlini, Valérie Suain, Zehra Yilmaz, Salwa Mansour, Isabelle Le Ber, Cécile Bouchard, Karelle Leroy, Alexandra Durr, Fabienne Clot, Marie Sarazin, Jean-Christophe Bier, Jean-Pierre Brion

**Affiliations:** 1 Laboratory of Histology, Neuroanatomy and Neuropathology, Faculty of Medicine, Université Libre de Bruxelles, ULB Neuroscience Institute, 808 route de Lennik, Bldg GE, B-1070 Brussels, Belgium; 2 Department of Neurology, Université Libre de Bruxelles, Hôpital Erasme, 808 route de Lennik, B-1070 Brussels, Belgium; 3 Sorbonne Université, Institut du Cerveau (ICM) - Paris Brain Institute, AP-HP, INSERM, CNRS, University Hospital Pitié-Salpêtrière, Paris, France; 4 Boulogne Billancourt, France; 5 Université Paris-Saclay, CEA, CNRS, Inserm, BioMaps, Orsay, France; 6 Unit of Neurology of memory and language, Groupe Hospitalier Universitaire Paris – Psychiatrie et Neurosciences, Hôpital Sainte Anne, Université de Paris, Paris, France and Université Paris-Saclay, CEA, CNRS, Inserm, BioMaps, 91401 Orsay, France

**Keywords:** Neurofibrillary tangles, Tau pathology, FTLD-tau, *MAPT*

## Main text

Tauopathies are neurodegenerative disorders characterized by hyperphosphorylated microtubule-associated protein tau (MAPT) forming protein aggregates. Over sixty dominantly inherited mutations in *MAPT* have been reported. These mutations cause frontotemporal lobar degeneration with tau-immunoreactive inclusions (FTLD-tau) whose symptom onset and survival are highly heterogeneous [4]. We describe here the genetic, clinical, neuropathological and biochemical characterization of a case of de novo *MAPT*-G335A mutation in a male patient.

Symptoms of poor organisation skills, attention and concentration difficulties appeared when the proband was 14 years old. A few years later, he developed anxiety, panic attacks and time obsession. Firstly, he was considered to be suffering from a schizophrenic disorder. Since behavioral and phasic disturbances progressivly appeared, he was referred to a department of neurology at the age of 24. By this time, MRI showed a frontotemporal atrophy and SPECT demonstrated an hypofixation in the same regions. The clinical evolution was marked by a progressive decline in cognitive functions, particularly in speech output, semantic memory and in executive functions, an hyperorality, an emotional blunting and a buccofacial apraxia with swallowing disturbance necessiting an institutionalisation in an appropiate facility when he was 25. He never developed major extrapyramidal features, except a dystonia of upper left arm and a slight upper arms rigidity probably of iatrogenic origin (i.e. neuroleptic drugs).

Sequencing of *MAPT* gene revealed a novel heterozygous mutation G335A due to G → C transition at the second base position in codon 335 in exon 12 (NM_005910.5: c.1004G > C p.Gly335Ala). The mutation was not found in his parents, thus strongly arguing for a de novo mutation. The proband died at 34, 20 years after the onset of symptoms. The proband’s brain was removed at autopsy, collected and stored in the brain bank of the LHNN (BB190052). One brain hemisphere was fixed in 10% buffered formalin. The other hemisphere was cut into coronal sections and kept at − 80 °C. A small piece of frontal cortex was fixed in 4% glutaraldehyde for transmission electron microscopy.

**Fig. 1:**
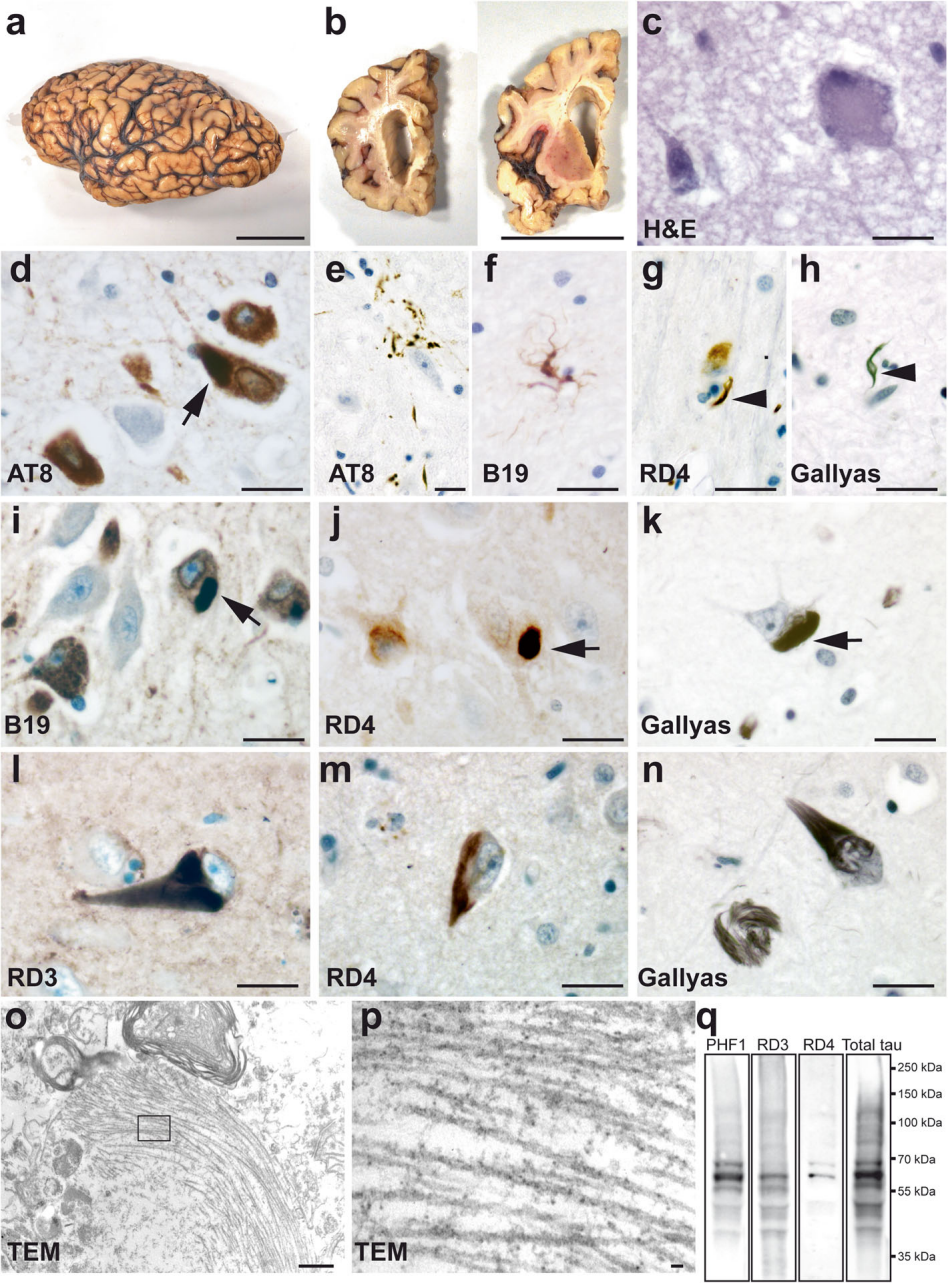
de novo mutation of *MAPT*-G335A led to severe brain atrophy with glial and neuronal tau pathology constituted of 3R and 4R tau. **a** Macroscopic pathology of FTLD-tau with *MAPT-*G335A mutation. The fixed left cerebral hemisphere showed a severe circumscribed atrophy of frontal lobe and anterior temporal lobe (with sparing of posterior superior gyrus). Motor and sensory gyri and occipital lobe were relatively spared. **b** Coronal sections of fixed brain. The frontal horn of the lateral ventricle was markedly dilated. **c** H&E reveals a ballooned neuron (right) and microvacuolation. **d-e** Immunostaining with AT8 (Thermo Scientific) (pS202/T205 tau) revealed numerous NFT in the pyramidal layer of the hippocampal CA1–2 sector (**d**). Occasionally there were astrocytic plaque-like appearance of tau-positive deposits in the striatum, thalamus and temporal cortex (**e**). **f** B19 (total tau)-positive astrocyte, reminiscent of tufted astrocytes were observed in the thalamus, frontal cortex and temporal cortex. **g** Coiled bodies (arrowhead) were immunolabelled by RD4 (clone 1E1/A6, Millipore) (4R tau). **h** Coiled bodies (arrowhead) were Gallyas positive. **i-k** Pyramidal neurons of the hippocampal CA2–3 sector immunostained for anti-tau B19 (**i**), RD4 (4R tau) (**j**) and Gallyas staining (**k**). Arrows show intraneuronal 4R tau positive globular and elongated inclusions (**d**, **i-k**). **l-n** NFT were immunostained with RD3 (clone 8E16/C11, Millipore, **l**) (3R tau) and RD4 (4R tau) (**m**). **n** Gallyas staining in CA2 sector shows a classical NFT (right) and Gallyas-positive intraneuronal threads (left). **o-p** Transmission electron microscopy showing a cytoplasmic aggregate of filaments in the frontal cortex. At higher magnification (**p**) of the inset in **o**, both straight filaments and PHF were observed. **o** 12,000x; **p** 30,000x. **q** Sarkosyl insoluble tau from the frontal cortex of the proband (*MAPT*-G335A) immunoblotted with PHF1, RD3, RD4 and total tau (A0024, Dako) antibodies. Sarkosyl insoluble tau is constituted of hyperphosphorylated 3R and 4R tau. Scale bars, 5 cm for **a**-**b**; 20 μm for **c**-**n**; 500 nm for **o** and 30 nm for **p**

The macroscopic examination showed a severe atrophy of the frontal and anterior temporal lobes (Fig. 1a) with a whole wet brain weight of 928 g. A marked dilatation of the frontal horn of the lateral ventricule was present (Fig. 1b). Neuropathological analysis was done on paraffin-embedded sections. In all regions examined, there were neurodegenerative changes consistent with a tauopathy (Table 1). Possible neuropathological comorbidity was excluded by immunohistochemistry for Aß, α-synuclein and TDP-43. Significant neuronal loss and microvacuolation were observed in the superior layers of frontal and temporal cortex, where some ballooned neurons were also observed (Fig. 1c). In the deep layers of frontal and temporal cortex, there were neurofibrillary tangles (NFT) with flame shaped or globular forms, pre-tangles, neuropil threads, grain-like neuropil threads that were immunolabelled for total tau (B19) [2] and phosphotau AT8. Some NFT were detected in the parietal cortex but not in occipital cortex. NFT were prominent in the hippocampal CA sector and in the subiculum (Fig. 1d). Some astrocytes contained granular or punctate tau-immunoreactive deposits reminiscent of astrocytic plaques and tufted astrocytes (Fig. 1e, f). Tau-imunoreactive astrocytes were predominantly 4R tau positive (Supplementary figure 1a-c, online resource). There were numbers of tau-positive coiled-bodies and axons in the white matter and in the deep cortical layers. The coiled bodies were labelled by 4R tau antibody and by Gallyas staining (Fig. 1g-h). Neuronal loss and NFT were prominent in substantia nigra. In the brainstem, tau positive lesions were detected in the pontine nuclei and in the olivary nuclei of the medulla. Some pyramidal neurons in hippocampal CA contained globular and elongated tau inclusions positive for 4R tau and Gallyas (Fig. 1d, i-k). NFT were detected by anti-3R and 4R tau antibodies (Fig. 1l, m). Some neurons had Gallyas-positive intraneuronal linear thread-like structures (Fig. 1n). GFAP positive gliosis was remarkable in the superior layers of the frontal cortex but less in the temporal cortex. A significant gliosis was observed in the sub-ependymal zone, especially at the level of the striatum and under the pia of the brainstem.

**Table 1:** Semi-quantitative assessment and regional distribution of tau pathology on brain sections immunolabelled with AT8. -: absent; +: occasional; ++: moderate number; +++: frequent number. NFT, neurofibrillary tangles

	Tau pathology			
Brain areas	NFT	Neuronal diffuse	Oligodendroglial	Astrocytic
Hippocampal pyramidal layer	+++	+++	++	+
Dentate gyrus	–	+	–	–
Subiculum	++	++	+	+
Transentorhinal cortex	+	+	+	+
Temporal cortex	++	++	++	+
Frontal cortex	++	+	++	+
Cingulate cortex	+	+	+	+
Visual cortex	–	–	–	–
Striatum	++	+	+++	+
Globus pallidus	++	+	+	+
Thalamus	++	+	+	+
Substantia nigra	+++	+	+	+
Pontine nuclei	++	+	+	+
Olivary Nuclei	–	+	–	–

The ultrastructural aspect of the tau inclusions was analysed on ultrathin sections by transmission electron microscopy (Fig. 1o). Fibrillar inclusions were composed of both straight and paired helical filament (PHF) (Fig. 1p).

The sarkosyl fractionation method was used to enrich the insoluble tau from frozen grey matter of the frontal cortex [1] (Fig. 1q). By immunobloting, the sarkosyl insoluble tau extracted from the proband showed three major bands of 60, 64 and 68 kDa and was constituted of both 3R and 4R tau isoforms.

This is the first report of the G335A mutation in exon 12 of *MAPT* that caused a strikingly early onset disease at 14 years old. Previously two *MAPT* mutations on the same amino acid have been reported to cause early onset frontotemporal dementia: the age of onset was 22 years old for *MAPT*-G335V [7] and 25.4 years old on average for *MAPT-*G335S [10]. Compared to other FTLD-tau-*MAPT* cases with an average age at onset of 49 years [4, 8], mutations at *MAPT*-G335 led to early onset and severe neuronal and glial tau pathology ultrastructurally composed of straight filaments and PHF in *MAPT*-G335A (this study) and in *MAPT*-G335S [10]. The *MAPT*-G335S mutation was associated to neuronal tau pathology (tau positive neurons, NFT, neuropil threads) and glial tau pathology (coiled bodies, tau positive astrocytes), similarly to what we observed here in the *MAPT*-G335A mutation. Neuronal inclusions in the present *MAPT*-G335A were labelled with both anti-3R tau and anti-4R tau antibodies, but 3R tau positive Pick bodies were not detected, in contrast with other *MAPT* mutations such as G272V [3] and L266V [5]. The immunobloting pattern of sarkosyl-insoluble tau in *MAPT*-G335A is similar to Alzheimer’s disease [9]; the presence of both 3R and 4R tau isoforms in the insoluble fraction was also observed in *MAPT*-G389R [6] and *MAPT*-L266V [5]. This *MAPT*-G335A mutation changes the third among the four invariant PGGG motif in the microtubule-binding region to PGGA, and is predicted to affect the binding of tau to microtubules. Indeed, G335V and G335S mutations were reported to strongly affect this binding [7, 10], a molecular effect that might lead to increased level of free tau and the assembly of tau into filaments.

## Supplementary information

**Additional file 1: Figure S1.** Tau positive astrocytes reminiscent of tufted astrocytes were predominantly detected by anti-4R tau RD4 antibody in the temporal cortex of MAPT-G335A brain. 4R tau isoform (green) is expressed in an astrocyte immunolabelled by the astrocyte marker GFAP (red). The mouse monoclonal anti-4R tau RD4 antibody (clone 1E1/A6, Millipore) and the rabbit polyclonal anti-GFAP antibody (Sigma G9269) were detected as previously described [1].

## Data Availability

Not applicable.

## Abbreviations

MAPT: Microtubule-associated protein tau
MRI: Magnetic Resonance Imaging
TDP-43: TAR DNA-binding protein 43
NFT: Neurofibrillary tangles
SPECT: Single-photon emission computed tomography
CA: Cornu Ammonis
3R tau: Three-repeat tau isoform
4R tau: Four-repeat tau isoform
PHF: Paired helical filament
