# Supplementary material for: de novo MAPT mutation G335A causes severe brain atrophy, 3R and 4R PHF-tau pathology and early onset frontotemporal dementia
Source: Acta Neuropathol Commun. 2020 Jun 29;8:94. doi: 10.1186/s40478-020-00977-8 (PMC7325098; doi:10.1186/s40478-020-00977-8)
Supplement: Supplementary file 1 — Additional file 1: Figure S1. Tau positive astrocytes reminiscent of tufted astrocytes were predominantly detected by anti-4R tau RD4 antibody in the temporal cortex of MAPT-G335A brain. 4R tau isoform (green) is expressed in an astrocyte immunolabelled by the astrocyte marker GFAP (red). The mouse monoclonal anti-4R tau RD4 antibody (clone 1E1/A6, Millipore) and the rabbit polyclonal anti-GFAP antibody (Sigma G9269) were detected as previously described [1]. [file 40478_2020_977_MOESM1_ESM.pdf]

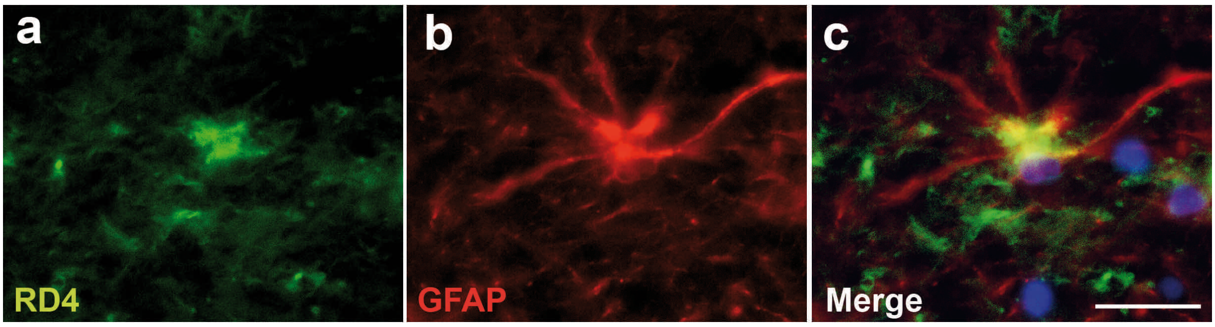

Figure S1

Supplementary figure 1

Tau positive astrocytes reminiscent of tufted astrocytes were predominantly detected by anti-4R tau RD4 in the temporal cortex of *MAPT*-G335A brain. 4-repeat (4R) tau isoform (*green*) is expressed in an astrocyte immunolabelled by the astrocyte marker GFAP (*red*). The mouse monoclonal anti-4R tau RD4 antibody (clone 1E1/A6, Millipore) and the rabbit polyclonal anti-GFAP antibody (Sigma G9269) were detected as previously described [1].

Reference:

1 Poncelet L, Ando K, Vergara C, Mansour S, Suain V, Yilmaz Z et al. (2019) A 4R tauopathy develops without amyloid deposits in aged cat brains. Neurobiol Aging 81: 200-212
